# Phase I/II Design for Selecting Subgroup‐Specific Optimal Biological Doses for Prespecified Subgroups

**DOI:** 10.1002/sim.10256

**Published:** 2024-10-18

**Authors:** Sydney Porter, Thomas A. Murray, Anne Eaton

**Affiliations:** ^1^ Division of Biostatistics University of Minnesota Minneapolis Minnesota USA

**Keywords:** Bayesian design, immunotherapy, optimal biological dose, spike and slab, subgroups

## Abstract

We propose a phase I/II trial design to support dose‐finding when the optimal biological dose (OBD) may differ in two prespecified patient subgroups. The proposed design uses a utility function to quantify efficacy‐toxicity trade‐offs, and a Bayesian model with spike and slab prior distributions for the subgroup effect on toxicity and efficacy to guide dosing and to facilitate identifying either subgroup‐specific OBDs or a common OBD depending on the resulting trial data. In a simulation study, we find the proposed design performs nearly as well as a design that ignores subgroups when the dose‐toxicity and dose‐efficacy relationships are the same in both subgroups, and nearly as well as a design with independent dose‐finding within each subgroup when these relationships differ across subgroups. In other words, the proposed adaptive design performs similarly to the design that would be chosen if investigators possessed foreknowledge about whether the dose‐toxicity and/or dose‐efficacy relationship differs across two prespecified subgroups. Thus, the proposed design may be effective for OBD selection when uncertainty exists about whether the OBD differs in two prespecified subgroups.

## 1 Introduction

Historically, phase I dose‐finding trials in cancer evaluated the toxicity of varying doses of a novel cytotoxic therapy with the aim of identifying the maximum tolerated dose (MTD), which is defined as the dose with a toxicity rate closest to some prespecified target rate, often between 0.1 and 0.3. For cytotoxic therapies, toxicity and efficacy are generally assumed to increase monotonically with dose and thus, the MTD is also the most effective tolerated dose. In contrast, for immunotherapies and targeted therapies, the probability of efficacy may plateau at higher doses and thus, a lower dose with similar efficacy may be preferable to the MTD [1, 2]. Therefore, trials evaluating newer classes of drugs such as immunotherapy and targeted therapies require consideration of toxicity and efficacy with the aim of identifying the optimal biological dose (OBD), which provides the most preferable toxicity and efficacy profile. In the context of solid tumors, toxicity and efficacy are often measured in a dichotomous manner. Dose‐limiting toxicities (DLTs) are generally defined as grade ≥3 non‐hematological or Grade 4 hematological toxicities, where grades are defined by the Common Terminology Criteria for Adverse Events (CTCAE) [3]. A commonly used definition for efficacy is the Response Evaluation Criteria in Solid Tumors (RECISTs) which categorizes response to treatment using the diameter of targeted lesions post‐treatment versus pretreatment. Efficacy is defined by observing a partial or complete response (≥30% decrease in the diameter of targeted lesions) while stable disease (<30% decrease to <20% increase in the diameter of targeted lesions) and progressive disease (≥20% increase in the diameter of targeted lesions) are indicators of no efficacy [4].

Our research is motivated by a phase I/II dose‐finding study at the University of Minnesota for a novel second‐line immunotherapeutic regimen which trains the immune system to target and kill glioblastoma cancer cells [5]. In patients with glioblastoma, immunotherapy can lead to cerebral inflammation and edema, which can lead to neurologic adverse events [6]. Previous data suggested that patients with larger tumors may have a higher risk of these neurologic adverse events, perhaps due to a stronger immune response and/or due to the tumor occupying a larger volume of the brain. Thus, it is hypothesized that toxicity rates may be higher in participants with larger tumors. If this is the case, counterintuitively, the optimal dose may be lower in participants with large tumors; however, very little data has been observed to support or refute this hypothesis. For practicality, we consider tumor size to be binary (large or small). To use established designs for identifying an OBD, the investigators could conduct dose‐finding independently within each tumor size group, which would be resource intensive, or conduct a trial that ignores potential subgroup differences and identifies a common OBD, which may result in sub‐optimal dosing of one or both subgroups in future trials. In response to these limitations of established designs, the Food and Drug Administration recently released guidance emphasizing the importance of accounting for covariates in dose finding [7]. There is a need for adaptive designs that facilitate efficient identification of OBDs in prespecified subgroups.

Several designs aim to find subgroup‐specific MTDs. Morita et al. detail a generalization of the continual reassessment method (CRM) using a hierarchical Bayesian dose‐toxicity model that borrows strength between subgroups under the assumption that the subgroups are exchangeable [8]. Salter et al. describe a variation of the time‐to‐event CRM (TITE‐CRM) which utilizes a two‐group likelihood to accommodate patient heterogeneity [9]. Horton et al. detail subgroup‐specific MTD identification in the context of a clinically meaningful group ordering [10]. O'Quigley and Iasonos describe models which can be used to bridge independent trials in two subgroups to facilitate efficient information use and improved trial performance [11]. Sub‐TITE, developed by Chapple and Thall, is a variation of the TITE‐CRM which utilizes spike and slab prior distributions to allow combining of subgroup terms in the model for the probability of toxicity if homogeneity is determined [12]. Additionally, Cotterill and Jaki have proposed a design that can find subgroup‐specific MTDs when a trial includes two prespecified subgroups [13]. This design uses a Bayesian dose‐toxicity model that places spike and slab prior distributions on subgroup‐specific regression coefficients. The spike and slab prior effectuates data driven variable selection such that when accumulating data support a common dose‐toxicity relationship across subgroups the subgroup‐specific regression coefficients are removed from the model and the pooled data from both subgroups is used to efficiently learn their common dose‐toxicity relationship. In this paper, we propose an extension of the Cotterill and Jaki design which supports dose‐finding based on efficacy‐toxicity trade‐offs, rather than toxicity alone. Our design uses a utility‐based approach to finding the OBD and allows for subgroup‐specific OBDs when the data suggest that there are different dose‐toxicity or dose‐efficacy relationships across the two prespecified subgroups, and for a common OBD when the data suggest the dose‐toxicity and dose‐efficacy relationships are the same across subgroups.

Designs that allow for dose‐finding based on toxicity and efficacy while accounting for patient covariates have been proposed previously [14, 15, 16]. Specifically, the design proposed by Thall, Nguyen, and Estey uses separate regression models for toxicity and efficacy with predictors that reflect dose and other prespecified covariates [14]. The main disadvantage of this design is that it requires historical data to set the prior distributions. Additionally, dose desirability is qualified using target contours whereas a utility‐based approach may be more intuitive. Cunanan and Koopmeiners present a hierarchical modeling approach to determine the optimal dose in subgroups based on maximizing the probability of efficacy without toxicity [15]. Although this design works well with a large number of subgroups, they advise against using their design with fewer than three subgroups. Similarly, Curtis et al. proposed a subgroup‐specific dose‐finding design which uses Bayesian clustering. This method performs best when there are at least two subgroups with different OBDs represented in the trial, but can underperform compared to a hierarchical design when all subgroups have the same OBD [17]. Lastly, other designs may be used for immunotherapies or targeted therapies when subgroups cannot be specified a priori. These designs can both identify subgroups within the data and determine subgroup‐specific OBDs, but require a much larger sample size [18, 19].

In Section 2, we present the toxicity and efficacy models underlying the proposed design and the guidelines dictating dose assignment during the trial. In Section 3, a simulation study is used to compare the operating characteristics of our proposed design to a trial that pools participants from both subgroups and a trial that conducts independent dose‐finding in each subgroup. The designs are compared under settings where the dose‐toxicity and/or dose‐efficacy relationships differ by subgroup and settings where they do not. Lastly, Section 4 presents a short discussion of the strengths and limitations for the proposed design and possible extensions.

## 2 Methods

In this section, we begin by describing the dose‐efficacy and dose‐toxicity models underlying our design and how a utility function is used to quantify the relative desirability of the four possible outcomes, that is, efficacy with and without toxicity, and no efficacy with or without toxicity. We then explain the additional rule‐based safeguards implemented in the design along with the general structure of the trial.

### 2.1 Dose‐Outcome Models

The proposed design requires one model for the dose‐efficacy relationship and one model for the dose‐toxicity relationship. Following Thall and Cooke, we let $\delta_{1},\ldots ,\delta_{J}$ denote the J doses under investigation with $d_{j}=\log\left(\delta_{j}\right)-J^{-1}\sum_{q=1}^{J}\log\left(\delta_{q}\right)$ as a standardized dose j=1,…,J [20]. Also, let k=0,1 reflect a binary indicator for the subgroup of interest, where the OBD may differ by k. For example, k could represent membership in the large tumor or small tumor subgroup. Letting $Y^{E}$ and $Y^{T}$, respectively denote binary indicators for the efficacy and toxicity outcomes, we are interested in the probabilities of efficacy and toxicity by dose and subgroup: $\pi_{k}^{E}\left(d\right)=P\left(Y^{E}=1|k,d\right)$ and $\pi_{k}^{T}\left(d\right)=P\left(Y^{T}=1|k,d\right)$. We assume the following dose‐efficacy model

(1) $$\log\left(\frac{\pi_{k}^{E}\left(d\right)}{1-\pi_{k}^{E}\left(d\right)}\right)=\beta_{E,0}+\beta_{E,1}d+\beta_{E,2}d^{2}+x_{k}\left(\beta_{E,3}+\beta_{E,4}d+\beta_{E,5}d^{2}\right)$$

with $x_{k}=2k-1$. Contrast coding of $x_{k}$ is used for more efficient posterior convergence compared to a 0–1 indicator [21].

This model is quadratic in the standardized dose to allow a range of dose‐efficacy relationships, including non‐monotone relationships and relationships that flatten out above a certain dose. Additionally, the interaction between dose and subgroup, and dose squared and subgroup allows flexibility in the case where the dose‐efficacy relationships in the two subgroups have different shapes.

In contrast, we assume the following dose‐toxicity model

(2) $$\log\left(\frac{\pi_{k}^{T}\left(d\right)}{1-\pi_{k}^{T}\left(d\right)}\right)=\beta_{T,0}+\beta_{T,1}d+x_{k}\left(\beta_{T,2}+\beta_{T,3}d\right)$$

This model reflects the expectation that the dose‐toxicity relationship is monotonically increasing as it does not include a quadratic dose effect. However, this model still allows the shape of the curve to differ by subgroup through the inclusion of the interaction between dose and subgroup.

We use spike and slab prior distributions for the subgroup effects in models 1 and 2 (i.e., $\beta_{E,3},\beta_{E,4},\beta_{E,5}$ and $\beta_{T,2},\beta_{T,3}$). The goal of the spike and slab prior distribution is to effectuate keeping effects related to subgroup in the models when the data support that the dose‐efficacy or dose‐toxicity relationship differs by subgroup. Otherwise, the spike and slab prior will effectuate a parsimonious model by setting some subgroup effects $\beta_{k,\ell }=0$. The spike and slab priors take the following form: $\beta_{k,\ell }|\gamma_{k,\ell }\overset{\mathrm{ind}.}{∼}\gamma_{k,\ell }N\left(b_{k,\ell },\sigma_{k,\ell }^{2}\right)+\left(1-\gamma_{k,\ell }\right)\times 0=\gamma_{k,\ell }N\left(b_{k,\ell },\sigma_{k,\ell }^{2}\right)$ where $\gamma_{k,\ell }\overset{\mathrm{ind}.}{∼}\text{Bernoulli}\left(p_{k,\ell }\right)$, for k=E,ℓ=3,4,5 and k=T,ℓ=2,3, and $\gamma_{k,\ell }$ is a latent indicator variable for whether $\beta_{k,\ell }$ is included in the model for outcome k. For the remaining coefficients, we use normal priors with the following form: $\beta_{k,\ell }\overset{\mathrm{ind}.}{∼}N\left(b_{k,\ell },\sigma_{k,\ell }^{2}\right)$, for k=E,ℓ=0,1,2 and k=T,ℓ=0,1. We will discuss hyperparameter specification in Section 2.3.

### 2.2 Utility: Definition and Estimation

Although our proposed model assumes toxicity and efficacy are independent random variables, our proposed design accounts for their joint distribution through a utility function [22]. The utility function assigns a utility $\nu_{a,b}$ to the outcome where $Y^{E}=a$ and $Y^{T}=b$ such that $0\leq \nu_{a,b}\leq 100$ for all a and b, and $\nu_{1,0}=100$ (the most desirable outcome) and $\nu_{0,1}=0$ (the least desirable outcome). In this way, the utility function reflects the relative desirability of each possible outcome. Because the chosen utility function will be context dependent and has direct implications for the OBD identified by the trial, it is recommended that a consensus is reached on the appropriate utility function by a group of clinicians who are familiar with the clinical and quality of life implications of these outcomes in the target patient population. Alternatively, a sensitivity analysis for the utility function could be conducted during or after the trial. Although the utility values are important, slight variations in the actual values are unlikely to cause major changes in the overall conclusion of the dose‐finding design as long as the ordering of the outcomes by utility remains the same [16].

The expected utility for a given dose $d_{j}$ in a given subgroup k is defined to be $U_{k}\left(d_{j}\right)=\sum_{a=0}^{1}\sum_{b=0}^{1}\nu_{a,b}\pi_{k}^{a,b}\left(d_{j}\right)$. The subgroup‐specific OBD is then defined as the admissible dose with the highest expected utility, for the given subgroup. During the trial, the admissible set, ${𝒜}_{k}$, is defined as those doses that satisfy Condition 1 and Condition 2a or 2b.

(1) $P\left(\pi_{k}^{T}\left(d_{j}\right)<{\bar{\pi }}_{T}\right)>p_{T}$.
(2a) $P\left(\pi_{k}^{E}\left(d_{j}\right)>{\underline{\pi }}_{E}\right)>p_{E}$ and $j\in {𝒥}_{\text{tried}}$.
(2b) $j=\max{𝒥}_{\text{tried}}+1$.

where ${\bar{\pi }}_{T}$ and ${\underline{\pi }}_{E}$ are the preset upper toxicity threshold and lower efficacy threshold, respectively, and $p_{T}$ and $p_{E}$ are the prespecified required level of posterior evidence needed to decide whether dose $d_{j}$ is a safe and effective dose. Common values for ${\underline{\pi }}_{E}$ and ${\bar{\pi }}_{T}$ are 0.3 or 0.35, but this will be dependent on the severity of the disease [13, 23, 24]. For feasible phase I/II sample sizes, common values for $p_{E}$ and $p_{T}$ are 0.05 or 0.1 [14, 24]. ${𝒥}_{\text{tried}}$ is the set of dose levels that have been administered so far during the trial. Upon conclusion of the trial, ${𝒜}_{k}$ is defined as all doses satisfying Conditions 1 and 2a.

During the trial and at the end of the trial, the expected utility of each dose for each subgroup, given the data, denoted ${\hat{U}}_{k}\left(d_{j}\right)$, is estimated assuming independence between efficacy and toxicity, that is,

(3) $${\hat{U}}_{k}\left(d_{j}\right)=\sum_{a=0}^{1}\sum_{b=0}^{1}\nu_{a,b}{\tilde{\pi }}_{k}^{E}\left(d_{j}\right){\tilde{\pi }}_{k}^{T}\left(d_{j}\right)$$

${\tilde{\pi }}_{k}^{E}\left(d_{j}\right)$ is estimated by calculating $P\left(Y^{E}=a|d_{j},k,\beta_{E}^{\left(m\right)}\right)$ for each of the m=1,…,M draws from the posterior distribution of $\beta_{E}=\left\{\beta_{E,0},\ldots ,\beta_{E,5}\right\}$ and estimating the maximum a posteriori (MAP) of that distribution [25]. ${\tilde{\pi }}_{k}^{T}\left(d_{j}\right)$ is determined similarly.

We make the simplifying independence assumption given that numerous authors have found that more parsimonious models often result in improved operating characteristics in the phase I/II setting even if the data generating process is partially misspecified [23, 24, 26, 27]. We will assess the performance of this simplifying assumption in our design using a simulation scenario where toxicity and efficacy are correlated. During the study, participants are assigned to the admissible dose that maximizes ${\hat{U}}_{k}\left(d_{j}\right)$ for their subgroup, and at the end of the trial, the OBD in each subgroup is selected as the admissible dose with the highest estimated expected utility ${\hat{U}}_{k}\left(d_{j}\right)$.

### 2.3 Prior Specification

For prior specification, we adapt the approach suggested by Thall et al. which algorithmically derives weakly informative prior hyperparameters which yield mean outcome probabilities and effective sample sizes (ESSs) close to elicited targets [28]. Under the working assumption that the dose‐outcome curves do not differ by subgroup, our dose‐outcome models are identical to the models described by Thall et al. and we can use their approach directly to derive hyperparameters for the coefficients unrelated to subgroup. Remaining consistent with our a priori assumption of no subgroup effect, we set $b_{T,2}=b_{T,3}=b_{E,3}=b_{E,4}=0$. We use $\sigma_{T,2}=\sigma_{T,3}=\sigma_{E,3}=\sigma_{E,4}=2.5$, which is consistent with current recommendations for prior variances for regression coefficients [29]. We recommend setting the prior inclusion probability, $p_{k,\ell }$, to 0.5 for all applicable k and ℓ to reflect the uncertainty about whether the dose‐outcome relationships differ by subgroup. Additional details on our prior specification approach can be found in the Supporting Information.

### 2.4 Trial Structure

**FIGURE 1:**
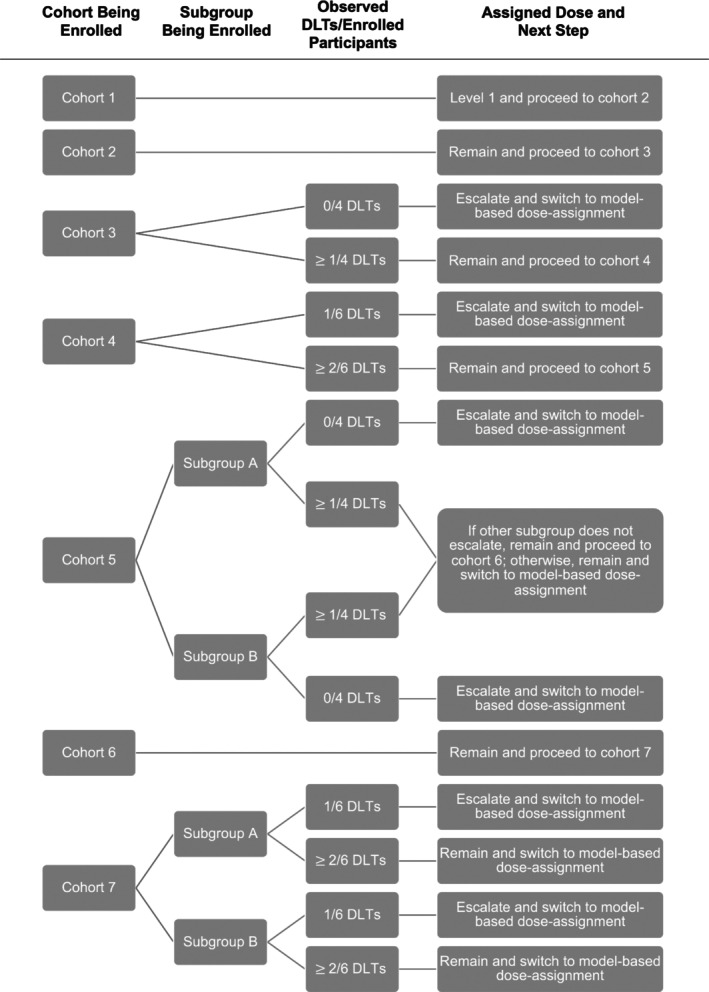
Flow‐diagram of trial's rule‐based run‐in period. The initial column indicates the cohort for which dose‐assignment is being determined. If dose‐assignment can differ by subgroup, the second column will split the dose‐assignment diagram by subgroup. The third column corresponds to the number of DLTs observed. Notation is given as “*x*/*y* DLTs,” where x is the cumulative number of observed DLTs overall or for the given subgroup, if subgroups are given in the second column, and y is the number of individuals enrolled overall, or by subgroup, if applicable. The notation “≥ *x*/*y* DLTs” is used to denote situations where ≥ *x* DLTs are observed out of y enrolled individuals. The fourth column will indicate whether the dose level should be escalated or remain at that which was previously assigned and will specify which dose‐assignment method should be used following enrollment of the current cohort at the specified dose level.

During the trial, participants are enrolled in cohorts of two, with one individual from each subgroup. Since the initial cohorts of the trial will be assigned doses based on little to no accrued data, we initially assign doses using a rule‐based approach that is meant to mimic the widely used 3 + 3 dose‐escalation design. The rule based run‐in, detailed in Figure 1, ensures the first dose assignment is the lowest dose; restricts the speed of dose escalation similar to the 3 + 3 approach, that is, the dose can only be escalated a single level between cohorts and dose‐escalation can only occur when no more than one out of six individuals treated at the current dose have experienced a DLT; keeps dose‐escalation from differing in the two subgroups early in the trial when there is insufficient information to determine if the OBD for the subgroups differ; and allows us to collect data and escalate the initial dose during a period of the trial where the dose‐assignment model would be unstable and give poor estimates.

Model‐based dose‐assignment proceeds as follows:

1. For each subgroup, calculate ${\hat{U}}_{k}\left(d_{j}\right)$, using the models described in Section 2.1 and Equation 3.
2. Determine ${𝒜}_{k}$ for each subgroup. For each subgroup, k,
  - If ${𝒜}_{k}=\emptyset$ do not enroll a participant from subgroup k.
  - If ${𝒜}_{k}\neq \emptyset$, enroll a participant from subgroup k and assign to dose $d_{j^{*}},j^{*}={\text{argmax}}_{j}{\hat{U}}_{k}\left(d_{j}\right),j\in {𝒜}_{k}$.

If ${𝒜}_{k}=\emptyset$ for both subgroups, the trial ends and we declare that none of the doses are acceptable for either subgroup. Otherwise, enrollment proceeds with model‐based dose‐assignment until the prespecified maximum number of cohorts are enrolled. If enrollment is closed in one group due to no admissible doses, cohorts of size one (one patient from the other subgroup) are enrolled. The subgroup that is closed for enrollment will be evaluated for re‐opening prior to the enrollment of each subsequent cohort since additional data in the open subgroup may justify re‐opening the closed subgroup.

Once enrollment is complete, the OBD for each subgroup is the admissible dose which has the largest subgroup‐specific estimated mean utility.

## 3 Simulation Study

### 3.1 Settings

Through simulation study, we compare our proposed method with two methods used in practice for finding an OBD when subgroups are present. In our simulations, the binary covariate of interest is tumor size (large versus small). The first method is a “pooled” approach where subgroups are completely ignored, that is, the trial is conducted and a common OBD is selected for both the large and small tumor groups based on the combined data. The second method is an “independent” approach where dose‐finding is conducted independently within each subgroup. In both comparison designs, the analysis models are equivalent to those in Equations 1 and 2 without the subgroup effect terms. All approaches use a maximum of 60 participants (30 per subgroup).

The set of possible doses is 𝒟={0.2,0.4,0.6,0.8,1} and dose is standardized as described in Section 2.1. Five thousand simulated trials were conducted for each scenario and approach. Posterior inference was performed using the BoomSpikeSlab R package [30] with a burn‐in period of 5000 draws followed by 15 000 draws for estimation. The utility function used for all scenarios assigns values to outcomes as follows: $\nu_{0,1}=0$, $\nu_{0,0}=40$, $\nu_{1,1}=60$, and $\nu_{1,0}=100$. In this case, we have deemed toxicity and efficacy to be more desirable than no toxicity and no efficacy implying that efficacy is more important than having no toxicity. We follow the recommended process for setting prior hyperparameters, outlined in Section 2.3 and the Supporting Information, using elicited mean outcome probabilities $\pi^{E}=\left(0.3,0.5,0.6,0.65,0.7\right)$ and $\pi^{T}=\left(0.05,0.1,0.15,0.2,0.25\right)$ and ESS =0.9. Details regarding the resulting hyperparameters for the primary simulation study can be found in the Supporting Information. Additionally, we explore the sensitivity of our design to alternative sets of elicited mean outcome probabilities, ESS and utility functions.

**FIGURE 2:**
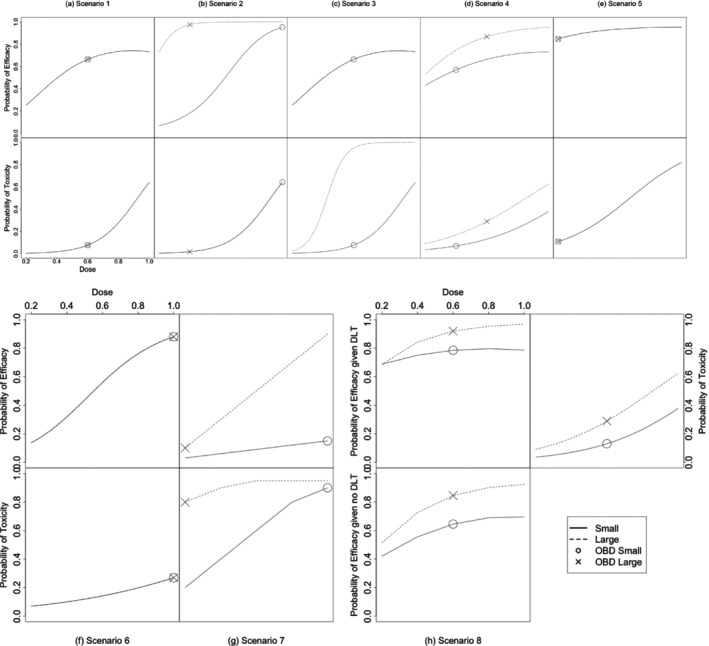
Dose‐efficacy curve, dose‐toxicity curve and subgroup‐specific OBD for eight simulation scenarios. For subfigures (a–g), the top panel is the dose‐efficacy curve and the bottom panel is the dose‐toxicity curve. Subfigure (h) gives the dose‐efficacy curves for each group conditional on whether a DLT was observed in addition to the marginal dose‐toxicity curve. The top left panel shows the probability of efficacy given a DLT is observed and the bottom panel shows the probability of efficacy given no DLT was observed. The top right panel describes the marginal dose‐toxicity relationship for Scenario 8.

Figure 2 shows the true dose‐efficacy curve, dose‐toxicity curve, and subgroup‐specific OBD for each of the simulation scenarios. The dose‐efficacy and dose‐toxicity models are of the same form as that described in Section 2.1 for Scenarios 1 through 6, but differ for Scenario 7. For Scenario 7, efficacy and toxicity probabilities are selected for each dose and subgroup without following a specific model form. In the first 7 scenarios, efficacy and toxicity are independent. In Scenario 8, the two outcomes are positively correlated.

The probability of efficacy and probability of toxicity thresholds for the simulation study are the same for all scenarios and were chosen based on threshold values used in papers investigating similar methodological problems [13, 15, 16]. The minimum acceptable probability of efficacy for each dose (${\underline{\pi }}_{E}$) is 0.3 while an unacceptable probability of toxicity (${\bar{\pi }}_{T}$) is 0.35 or higher. Posterior evidence thresholds ($p_{E}$ and $p_{T}$) were set at 0.1, which is commonly used in this setting [20]. Doses that fall outside of these acceptability thresholds for each of the scenarios are indicated in Table S3. The true OBDs shown in Figure 2 are defined as the doses with the maximum utility of the doses which meet these two thresholds.

### 3.2 Results

**FIGURE 3:**
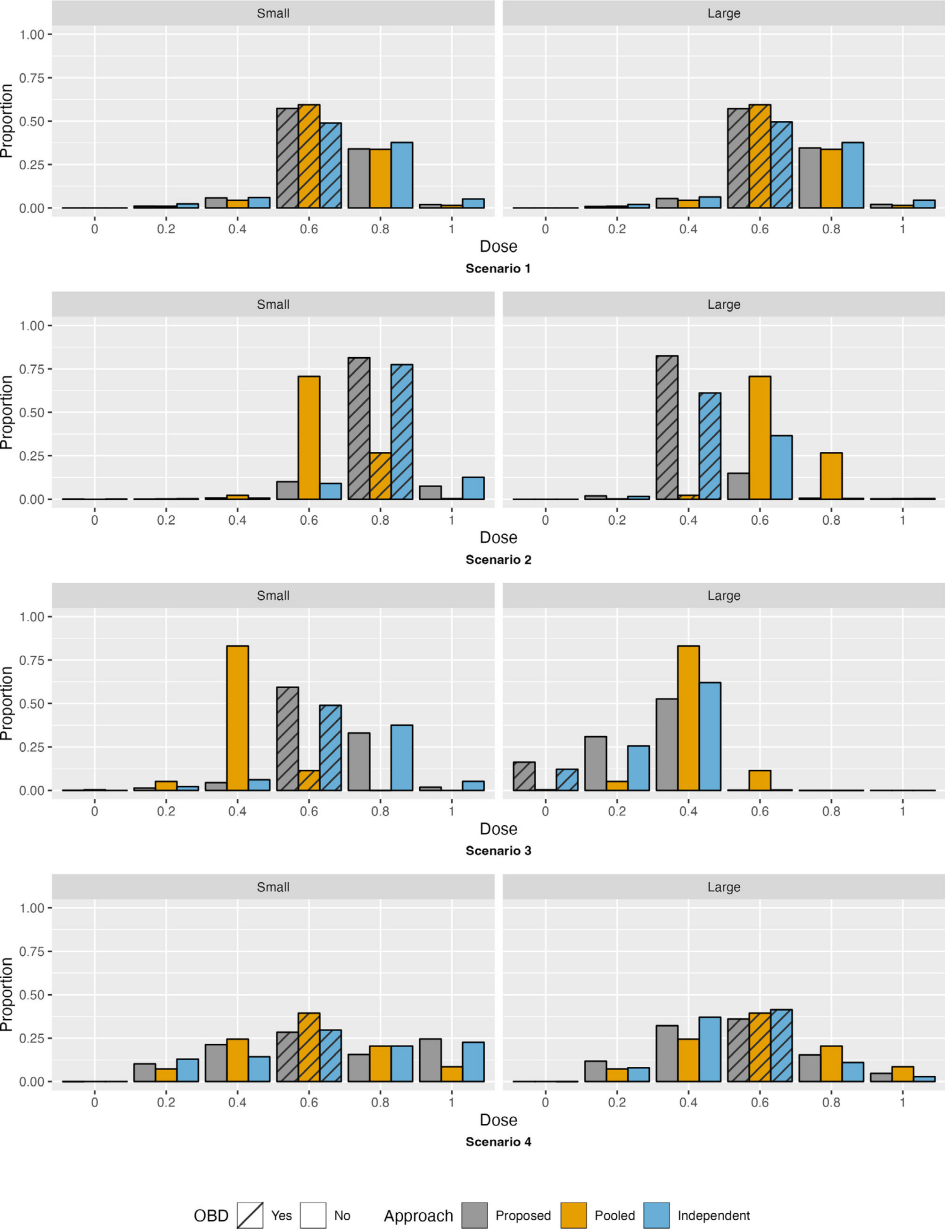
Proportion of time each dose was selected for each subgroup and approach, Scenarios 1–4. Each scenario is presented in a different row. The gray bars show results from our proposed approach, the orange bars show results from the pooled approach and the blue bars show results from the independent approach. For each scenario, the small tumor subgroup results are given on the left and the large tumor subgroup results are given on the right. A recommended dose of 0 means that no OBD has been declared because no dose meets the toxicity and efficacy thresholds.

**FIGURE 4:**
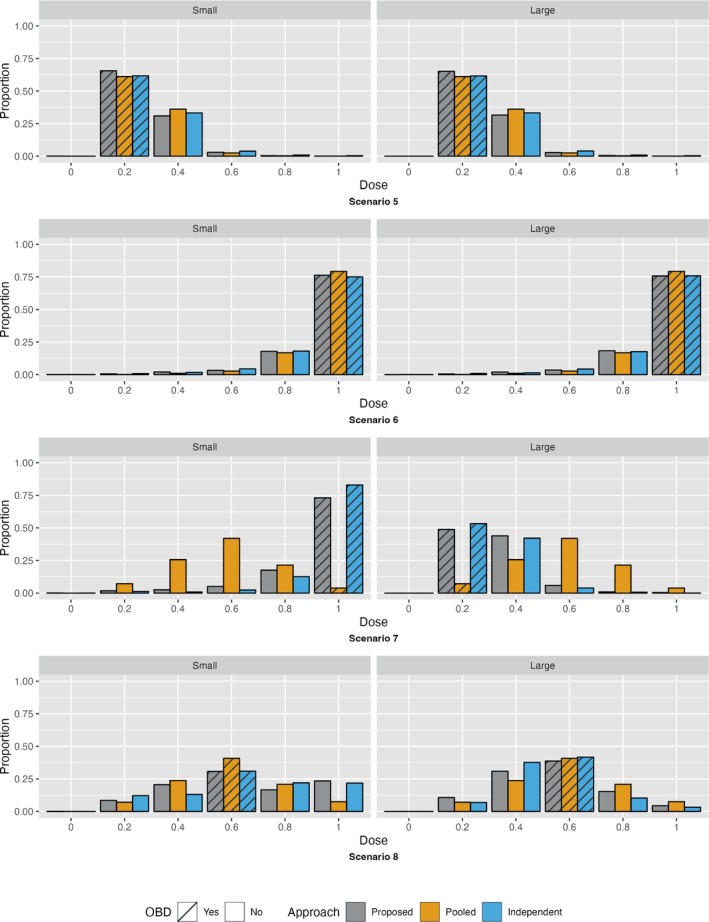
Proportion of time each dose was selected for each subgroup and approach, Scenarios 5–8. Each scenario is presented in a different row. The gray bars show results from our proposed approach, the orange bars show results from the pooled approach and the blue bars show results from the independent approach. For each scenario, the small tumor subgroup results are given on the left and the large tumor subgroup results are given on the right. A recommended dose of 0 means that no OBD has been declared because no dose meets the toxicity and efficacy thresholds.

Figures 3, 4, and Figure S1 present the probability of selecting each dose as the OBD, in two different ways, for each of the 8 scenarios and three dose‐finding approaches. Numerical values for the probability of selection as the OBD for each dose can be found in the Supporting Information along with the probability that the OBD was selected correctly for both subgroups and the probability that the OBDs selected were different in the two subgroups. The probability that the OBD was selected correctly for both subgroups for the proposed approach was generally somewhere in between the probabilities corresponding to the other two approaches for each scenario.

As expected, when the subgroups have the same dose‐outcome curves (Scenarios 1, 5, and 6), the pooled approach outperforms the independent approach in terms of percent correct selection of the OBD. Notably, the proposed approach also outperforms the independent approach and performs well when compared to the pooled approach. Likewise, when at least one of the dose‐efficacy and dose‐toxicity curves differs for the two subgroups leading to different true OBDs for the two subgroups (Scenarios 2, 3 and 7), the pooled approach performs the worst of the three designs and has a low probability of selecting the correct OBD for either subgroup. Instead, the selected OBD is typically somewhere in between the two true OBDs. Additionally, the proposed approach performs similarly to the more efficient comparison design, the independent approach. Of note, in Scenario 3, all three approaches have a relatively low probability of deciding that none of the doses are acceptable for the large tumor subgroup; instead, 0.2 or 0.4 are often selected as the OBD. This is likely due to the fact that while 0.2 and 0.4 are unacceptable doses in the large tumor group due to probability of efficacy below the efficacy threshold (Dose 0.2) and probability of toxicity above the toxicity threshold (Dose 0.4), the probabilities are only slightly above or below the respective thresholds. Lastly, when the dose‐efficacy and/or dose‐toxicity curves differ for the two subgroups but the true OBDs are the same (Scenarios 4 and 8), all three designs have difficulty identifying the true OBD. In terms of percent correct selection, the pooled approach seems to perform the best in the small tumor group, but the independent approach performs the best in the large tumor subgroup. Our proposed approach performs marginally worse than these two approaches for each subgroup. Since all three designs have similar performance in Scenarios 4 and 8, we can attribute the low percent correct selection of the OBD in Scenario 8 to the dose‐outcome curves used for the simulation rather than the correlated outcomes.

The average number of participants enrolled for trials was similar across designs, with the proposed method resulting in a maximum average reduction of two participants. This was accomplished without any substantial increases in the average proportion of DLTs overall (Table S4). Additionally, the number of participants treated at the OBD for the proposed approach and the optimal approach was nearly the same in most scenarios and in some cases the number of participants treated at the OBD was greater for the proposed approach than the optimal approach (Figures S2 and S3). In scenarios where the OBD was the same for both subgroups, the number of individuals who were overdosed was similar across all three approaches; however, when the OBD for one of the groups was a low dose and the two OBDs differed, the proposed approach and the independent approach overdosed substantially fewer individuals than the pooled approach. Results are presented in the Supporting Information.

Table S2 demonstrates how differing elicited mean outcome probabilities and ESS impact the resulting hyperparameters. Simulation results in Table S3 show how the elicited mean outcome probabilities, ESS, and utility function impact the probability of selecting each dose as the OBD. If the magnitude of the elicited ESS is moderate, then the impact of small variations in the elicited mean outcome probabilities on design performance will be minimal. Using an ESS less than 0.9 may not allow adequate exploration of the doses during the trial (see Scenarios 6 and 7 results) while using an ESS slightly greater than 0.9 has minimal effects on design performance.

Sensitivity analyses for cohort composition and the rule‐based run‐in were performed. For each scenario, the impact of unrestricted cohort composition (i.e., no requirement for cohorts to have one individual from each subgroup) on the distribution of the selected OBD is minimal. Additionally, design operating characteristics were similar when omitting the rule‐based run‐in in favor of complete model‐based dose‐assignment; however, the average proportion of DLTs increased, indicating a greater propensity to overdose (see results in the Supporting Information).

## 4 Discussion

We proposed a Bayesian adaptive dose‐finding design that effectively identifies subgroup‐specific OBDs or a common OBD when appropriate. Our proposed design performs similar to the design that would have been selected had one known the true dose‐efficacy and dose‐toxicity curves in advance. In most early phase clinical trials, very few participants have been treated with the investigational agent when the trial is designed. Investigators may hypothesize that toxicity or efficacy differs by a patient characteristic and thus that the optimal dose may differ, but their level of confidence would likely be too low to plan separate dose‐finding studies for different types of participants. Thus, a design like ours is highly desirable as it can identify subgroup‐specific OBDs if the data indicates they are needed but performs almost as well as a single trial ignoring subgroup if dose‐outcome relationships are the same in both subgroups.

In addition to selecting OBDs for future clinical trials, we may want to use the trial data to draw a conclusion about whether or not subgroup affects toxicity and/or efficacy. Cotterill and Jaki propose setting a threshold value for the posterior inclusion probability and concluding a subgroup effect exists if the probability is above the threshold [13]; however, we found that this method did not perform well when using our design because the posterior inclusion probabilities are heavily influenced by the prior inclusion probabilities (results not shown). Additionally, our proposed design, like other model‐based dose‐finding designs, prioritizes good estimates of the outcome probabilities at doses near the OBD, and only needs to properly order the utility of the doses to identify the correct OBD. Using estimated model coefficients may result in poor estimates of the outcome probabilities for doses far from the OBD due to parametric extrapolation.

The proposed design closes enrollment based on a prespecified number of cohorts when at least one of the subgroups has an admissible dose. Though this could result in an imbalance between subgroups if enrollment is closed for one subgroup and later reopened, subgroups were rarely closed in our simulations and large imbalances were not observed. If this is a concern, enrollment in the trial could be continued until subgroup‐specific maximum sample sizes have been reached.

The modeling approach used in the proposed design uses independent spike and slab prior distributions for each of the subgroup specific coefficients. An alternative approach, which we do not explore in this paper, would be the use of grouped spike and slab prior distributions. Under this framework, a common spike and slab coefficient would be used across all subgroup‐specific effects within a dose‐toxicity or dose‐efficacy model. This would restrict the model form, but could improve efficiency in that fewer parameters need to be estimated.

Lastly, our motivating trial has a binary covariate that may impact both the toxicity and efficacy of a dose; however, a continuous covariate could easily be included in place of the binary covariate using the same framework. This would allow for personalized OBDs based on the covariate, but would require enrollment of individuals across the whole range of the covariate and may require a larger sample size to ensure reliable estimation of the OBD across the range of the covariate.

## Conflicts of Interest

The authors declare no conflicts of interest.

## Supporting information


Data S1.

## Data Availability

Data sharing not applicable to this article as no datasets were generated or analysed during the current study.
