# Supplementary material for: Phase I/II Design for Selecting Subgroup‐Specific Optimal Biological Doses for Prespecified Subgroups
Source: Stat Med. 2024 Oct 18;43(28):5401–11. doi: 10.1002/sim.10256 (PMC11586896; doi:10.1002/sim.10256)
Supplement: Supplementary file 1 — Data S1. [file SIM-43-5401-s001.pdf]

Received: Added at production | Revised: Added at production | Accepted: Added at production
DOI: xxx/xxxx

ARTICLE TYPE

Supporting information for "Phase I/II design for selecting subgroup-specific optimal biological doses for pre-specified subgroups"

Sydney Porter* | Thomas A. Murray | Anne Eaton

[1]Division of Biostatistics, University of Minnesota, Minnesota, USA

Correspondence
*Sydney Porter, Division of Biostatistics, University of Minnesota, 2221 University Avenue SE, Suite 200, Minneapolis, MN 55414. Email: bens0643@umn.edu

Supporting Information

This document contains details on hyperparameter formulation; an illustration of the impact of variations of elicited prior information on the resulting prior hyperparameters; a simulation study to determine the impact of variations of elicited prior information on design operating characteristics; further details on the simulation study and additional figures presenting design operating characteristics for the simulation study presented in the main paper; and sensitivity analyses for unrestricted subgroup combinations within enrollment cohorts and omission of the rule-based run-in.

1 | PRIOR SPECIFICATION

1.1 | Hyperparameter Formulation

As in the approach of Thall et al.[1], we make the simplifying assumption that $b_{E,2} = b_{E,5} = 0$ and $\sigma_{E,2} = \sigma_{E,5} = 0.2$. This reduces the number of parameters that need to be calibrated and will lead to a more parsimonious model unless the data suggest otherwise. Next, under a working assumption that the dose-outcome curves do not differ by subgroup, our dose-outcome models are identical to the models described by Thall et al., e.g., $\log\left(\frac{\pi_k^T(d)}{1-\pi_k^T(d)}\right) = \beta_{T,0} + \beta_{T,1} d$ for the toxicity model. To calibrate the hyperparameters for $\beta_{T,0}, \beta_{T,1}, \beta_{E,0}$ and $\beta_{E,1}$, we first elicit sets of mean outcome probabilities from clinicians. The elicited probabilities will be context-dependent and must exceed the pre-specified efficacy threshold and are below the pre-specified toxicity threshold for all doses, e.g., $\boldsymbol{\pi}^{\mathbf{E}} = (0.3, 0.5, 0.6, 0.65, 0.7)$ and $\boldsymbol{\pi}^{\mathbf{T}} = (0.05, 0.1, 0.15, 0.2, 0.25)$ given a pre-specified toxicity threshold of 0.35 and a pre-specified efficacy threshold of 0.3. Additionally, the mean toxicity probabilities should be

increasing, while the mean efficacy probabilities should be non-decreasing with dose to encourage dose exploration during the trial. Next, we select the ESS to use for each model parameter. Thall et al. recommend using the same ESS for all model parameters and using an ESS between 0.3 and 1.5, with 0.9 often working well. Finally, remaining consistent with our *a priori* assumption of no subgroup effect, we set $b_{T,2} = b_{T,3} = b_{E,3} = b_{E,4} = 0$. We use $\sigma_{T,2} = \sigma_{T,3} = \sigma_{E,3} = \sigma_{E,4} = 2.5$, which is consistent with current recommendations for prior variances for regression coefficients.[2] We recommend setting the prior inclusion probability, $p_{k,\ell}$, to 0.5 for all applicable k and ℓ to reflect the uncertainty about whether the dose-outcome relationships differ by subgroup.

1.2 | Impact of Elicited Prior Information

Supplemental Table 1 presents five different elicitations that will be used to demonstrate the impact of each of the elicited parameters on the prior hyperparameters and design performance.

Elicitation	$\boldsymbol{\pi}^{\mathbf{E}}$	$\boldsymbol{\pi}^{\mathbf{T}}$	ESS	$(\nu_{0,1}, \nu_{0,0}, \nu_{1,1}, \nu_{1,0})$
1	(0.3, 0.5, 0.6, 0.65, 0.7)	(0.05, 0.1, 0.15, 0.2, 0.25)	0.9	(0, 40, 60, 100)
2	(0.3, 0.5, 0.7, 0.9, 1)	(0.1, 0.15, 0.2, 0.25, 0.35)	0.9	(0, 40, 60, 100)
3	(0.3, 0.5, 0.6, 0.65, 0.7)	(0.05, 0.1, 0.15, 0.2, 0.25)	0.5	(0, 40, 60, 100)
4	(0.3, 0.5, 0.6, 0.65, 0.7)	(0.05, 0.1, 0.15, 0.2, 0.25)	1.5	(0, 40, 60, 100)
5	(0.3, 0.5, 0.6, 0.65, 0.7)	(0.05, 0.1, 0.15, 0.2, 0.25)	0.9	(0, 20, 85, 100)

Supplemental Table 1 Elicited parameters for each of 5 elicitation settings. $\boldsymbol{\pi}^{\mathbf{E}}$ and $\boldsymbol{\pi}^{\mathbf{T}}$ are the elicited prior means for efficacy and toxicity, respectively; ESS is the elicited effective sample size; and $(\nu_{0,1}, \nu_{0,0}, \nu_{1,1}, \nu_{1,0})$ is the elicited set of utilities.

The impact of each elicitation setting on the prior hyperparameters is shown in Supplemental Table 2.

Elicitation	Design	$\mathbf{b_E}$	$\boldsymbol{\sigma}_\mathbf{E}$	$\mathbf{b_T}$	$\boldsymbol{\sigma}_\mathbf{T}$	Prior P(Efficacy)	Prior P(Toxicity)	Prior P(OBD)
1	Proposed	(0.57, 1.74, 0, 0, 0, 0)	(2.36, 2.12, 0.2, 2.5, 2.5, 0.2)	(-4.44, 1.03, 0, 0)	(2.62, 2.52, 2.5, 2.5)	(0.41, 0.51, 0.58, 0.63, 0.65)	(0.15, 0.13, 0.15, 0.18, 0.21)	(0.14, 0.17, 0.20, 0.24, 0.25)
	Comparison	(0.57, 1.74, 0)	(2.36, 2.12, 0.2)	(-4.44, 1.03)	(2.62, 2.52)	(0.38, 0.52, 0.61, 0.67, 0.70)	(0.09, 0.07, 0.09, 0.12, 0.15)	(0.10, 0.14, 0.21, 0.26, 0.28)
2	Proposed	(1.17, 4.85, 0, 0, 0, 0)	(2.51, 2.63, 0.2, 2.5, 2.5, 0.2)	(-7.33, -4.57, 0, 0)	(4.00, 3.95, 2.5, 2.5)	(0.25, 0.49, 0.68, 0.78, 0.83)	(0.33, 0.12, 0.06, 0.04, 0.04)	(0.02, 0.07, 0.17, 0.32, 0.43)
	Comparison	(1.17, 4.85, 0)	(2.51, 2.63, 0.2)	(-7.33, -4.57)	(4.00, 3.95)	(0.19, 0.48, 0.73, 0.84, 0.89)	(0.30, 0.09, 0.04, 0.02, 0.02)	(0.01, 0.03, 0.14, 0.32, 0.50)
3	Proposed	(0.66, 3.41, 0, 0, 0, 0)	(4.19, 4.25, 0.2, 2.5, 2.5, 0.2)	(-30.72, -2.09, 0, 0)	(25.16, 25.09, 2.5, 2.5)	(0.35, 0.48, 0.59, 0.65, 0.68)	(0.21, 0.12, 0.11, 0.12, 0.14)	(0.10, 0.13, 0.20, 0.27, 0.31)
	Comparison	(0.66, 3.41, 0)	(4.19, 4.25, 0.2)	(-30.72, -2.09)	(25.16, 25.09)	(0.33, 0.48, 0.60, 0.67, 0.71)	(0.20, 0.12, 0.11, 0.12, 0.14)	(0.09, 0.12, 0.20, 0.28, 0.32)
4	Proposed	(0.05, 1.73, 0, 0, 0, 0)	(1.75, 1.72, 0.2, 2.5, 2.5, 0.2)	(-3.96, 0.00, 0, 0)	(2.30, 2.26, 2.5, 2.5)	(0.36, 0.45, 0.53, 0.58, 0.61)	(0.22, 0.16, 0.15, 0.17, 0.18)	(0.11, 0.15, 0.20, 0.26, 0.27)
	Comparison	(0.05, 1.73, 0)	(1.75, 1.72, 0.2)	(-3.96, 0.00)	(2.30, 2.26)	(0.29, 0.43, 0.55, 0.62, 0.67)	(0.14, 0.09, 0.09, 0.10, 0.11)	(0.07, 0.11, 0.19, 0.29, 0.34)
5	Proposed	(0.57, 1.74, 0, 0, 0, 0)	(2.36, 2.12, 0.2, 2.5, 2.5, 0.2)	(-4.44, 1.03, 0, 0)	(2.62, 2.52, 2.5, 2.5)	(0.41, 0.51, 0.58, 0.63, 0.65)	(0.15, 0.13, 0.15, 0.18, 0.21)	(0.14, 0.16, 0.20, 0.25, 0.26)
	Comparison	(0.57, 1.74, 0)	(2.36, 2.12, 0.2)	(-4.44, 1.03)	(2.62, 2.52)	(0.38, 0.52, 0.61, 0.67, 0.70)	(0.09, 0.07, 0.09, 0.12, 0.15)	(0.10, 0.13, 0.20, 0.27, 0.30)

Supplemental Table 2 Implied hyperparameters and prior probabilities for each of 5 elicitation settings. For each elicitation, hyperparameters and prior probabilities are given for the proposed and comparison design models, since the comparison designs assume no subgroup effect. $\mathbf{b_E} = (b_{E,0}, b_{E,1}, b_{E,2}, b_{E,3}, b_{E,4}, b_{E,5})$ and $\boldsymbol{\sigma}_\mathbf{E} = (\sigma_{E,0}, \sigma_{E,1}, \sigma_{E,2}, \sigma_{E,3}, \sigma_{E,4}, \sigma_{E,5})$ for the proposed design. Likewise, $\mathbf{b_T} = (b_{T,0}, b_{T,1}, b_{T,2}, b_{T,3})$ and $\boldsymbol{\sigma}_\mathbf{T} = (\sigma_{T,0}, \sigma_{T,1}, \sigma_{T,2}, \sigma_{T,3})$ for the proposed design. For the comparison designs, $\mathbf{b_E} = (b_{E,0}, b_{E,1}, b_{E,2})$, $\boldsymbol{\sigma}_\mathbf{E} = (\sigma_{E,0}, \sigma_{E,1}, \sigma_{E,2})$, $\mathbf{b_T} = (b_{T,0}, b_{T,1})$ and $\boldsymbol{\sigma}_\mathbf{T} = (\sigma_{T,0}, \sigma_{T,1})$. “Prior P(Efficacy)” gives the implied prior probability of efficacy for each dose while “Prior P(Toxicity)” gives the implied prior probability of toxicity for each dose. Lastly, “Prior P(OBD)” gives the implied prior predictive probability of each dose level 1 through 5 being the OBD.

The resulting design performance for the proposed design using elicitation settings 1-5 is given in Supplemental Table 3. Elicitation 1 was used for simulations in the main text.

				Recommended dose for Small Tumor Group						Recommended dose for Large Tumor Group					
Scenario	Elicitation	P(Different OBDs)	P(Both OBDs Selected)	0	0.2	0.4	0.6	0.8	1	0	0.2	0.4	0.6	0.8	1
	1	0.27	0.46	0.00	0.01	0.06	0.57^X	0.34	0.02	0.00	0.01	0.05	0.57^X	0.35	0.02
	2	0.27	0.40	0.00	0.00	0.03	0.50^X	0.43	0.03	0.00	0.00	0.04	0.51^X	0.42	0.03
1	3	0.21	0.59	0.00	0.05	0.03	0.69^X	0.22	0.00	0.00	0.05	0.04	0.69^X	0.23	0.00
	4	0.30	0.40	0.00	0.01	0.06	0.51^X	0.39	0.03	0.00	0.01	0.06	0.52^X	0.38	0.03
	5	0.24	0.69	0.00	0.01	0.01	0.10	0.80^X	0.08	0.00	0.01	0.01	0.10	0.80^X	0.08
	1	0.98	0.68	0.00	0.00	0.01	0.10	0.81^X	0.08	0.00	0.02	0.82^X	0.15	0.01	0.00
	2	0.98	0.69	0.00	0.00	0.00	0.07	0.83^X	0.10	0.00	0.01	0.84^X	0.15	0.00	0.00
2	3	0.90	0.42	0.01	0.00	0.01	0.32	0.65^X	0.01	0.00	0.02	0.66^X	0.32	0.00	0.00
	4	0.98	0.65	0.00	0.00	0.00	0.08	0.82^X	0.10	0.00	0.03	0.80^X	0.16	0.01	0.00
	5	0.98	0.36	0.00	0.00	0.01	0.03	0.79^X	0.17	0.00	0.01	0.53	0.45^X	0.01	0.00
	1	0.97	0.07	0.00	0.01	0.04	0.59^X	0.33	0.02	0.16^X	0.31	0.53	0.00	0.00	0.00
	2	0.98	0.08	0.00	0.01	0.04	0.53^X	0.39	0.04	0.23^X	0.23	0.53	0.00	0.00	0.00
3	3	0.90	0.11	0.01	0.07	0.09	0.62^X	0.21	0.01	0.21^X	0.27	0.51	0.00	0.00	0.00
	4	0.98	0.06	0.00	0.01	0.04	0.55^X	0.38	0.02	0.15^X	0.31	0.54	0.00	0.00	0.00
	5	0.99	0.13	0.00	0.01	0.01	0.11	0.71^X	0.15	0.17^X	0.19	0.64	0.00	0.00	0.00
	1	0.66	0.13	0.00	0.10	0.21	0.28^X	0.16	0.24	0.00	0.12	0.32	0.36^X	0.15	0.05
	2	0.72	0.12	0.00	0.06	0.16	0.26^X	0.18	0.33	0.00	0.07	0.28	0.40^X	0.20	0.06
4	3	0.55	0.14	0.00	0.19	0.23	0.35^X	0.13	0.10	0.00	0.24	0.39	0.29^X	0.06	0.02
	4	0.69	0.12	0.00	0.08	0.20	0.27^X	0.17	0.28	0.00	0.10	0.29	0.37^X	0.19	0.05
	5	0.70	0.08	0.00	0.09	0.03	0.12	0.21^X	0.55	0.00	0.06	0.07	0.35^X	0.39	0.13
	1	0.22	0.55	0.00	0.66^X	0.31	0.03	0.00	0.00	0.00	0.65^X	0.32	0.03	0.01	0.00
	2	0.27	0.36	0.00	0.47^X	0.48	0.04	0.01	0.00	0.00	0.47^X	0.48	0.04	0.01	0.00
5	3	0.20	0.56	0.00	0.65^X	0.33	0.02	0.00	0.00	0.00	0.65^X	0.33	0.01	0.00	0.00
	4	0.21	0.60	0.00	0.69^X	0.27	0.03	0.01	0.00	0.00	0.69^X	0.27	0.03	0.01	0.00
	5	0.39	0.28	0.00	0.27	0.43^X	0.26	0.03	0.01	0.00	0.28	0.42^X	0.26	0.03	0.00
	1	0.21	0.67	0.00	0.01	0.02	0.03	0.18	0.76^X	0.00	0.00	0.02	0.04	0.18	0.76^X
	2	0.17	0.75	0.00	0.00	0.01	0.02	0.14	0.83^X	0.00	0.00	0.01	0.02	0.14	0.83^X
6	3	0.34	0.32	0.01	0.01	0.04	0.16	0.36	0.41^X	0.01	0.01	0.04	0.18	0.35	0.41^X
	4	0.18	0.75	0.00	0.00	0.02	0.02	0.12	0.83^X	0.00	0.00	0.01	0.02	0.13	0.83^X
	5	0.08	0.92	0.00	0.00	0.02	0.00	0.02	0.95^X	0.00	0.00	0.01	0.00	0.03	0.95^X
	1	0.97	0.34	0.00	0.02	0.03	0.05	0.18	0.73^X	0.00	0.49^X	0.44	0.06	0.01	0.00
	2	0.98	0.29	0.00	0.01	0.01	0.04	0.15	0.80^X	0.00	0.37^X	0.53	0.07	0.01	0.01
7	3	0.92	0.25	0.01	0.06	0.06	0.10	0.28	0.49^X	0.00	0.55^X	0.41	0.03	0.01	0.00
	4	0.98	0.37	0.00	0.02	0.02	0.04	0.14	0.78^X	0.00	0.49^X	0.43	0.06	0.01	0.01
	5	0.98	0.50	0.00	0.01	0.01	0.00	0.05	0.93^X	0.00	0.23	0.54^X	0.20	0.03	0.01
	1	0.66	0.15	0.00	0.09	0.21	0.31^X	0.17	0.23	0.00	0.11	0.31	0.39^X	0.15	0.04
	2	0.71	0.13	0.00	0.05	0.16	0.27^X	0.19	0.33	0.00	0.06	0.27	0.41^X	0.20	0.06
8	3	0.55	0.15	0.00	0.17	0.22	0.37^X	0.15	0.09	0.00	0.22	0.41	0.29^X	0.06	0.02
	4	0.70	0.12	0.00	0.08	0.18	0.27^X	0.18	0.28	0.00	0.08	0.30	0.38^X	0.19	0.05
	5	0.71	0.12	0.08	0.08	0.03	0.11	0.22^X	0.56	0.00	0.05	0.06	0.37^X	0.40	0.12

Supplemental Table 3 The probability of recommending each dose for each scenario and elicitation using the proposed design. “X" denotes the OBD. A shaded gray box indicates probability of efficacy less than the efficacy threshold and/or probability of toxicity greater than the toxicity threshold. A recommended dose of 0 means that no OBD has been declared because no dose meets the toxicity and efficacy thresholds.

2 | SIMULATION STUDY

2.1 | Settings Details

2.1.1 | Prior Distributions

We follow the recommended process for setting prior hyperparameters, outlined in Section 2.3 of the main paper, using elicited prior means of $\boldsymbol{\pi}^{\mathbf{E}} = (0.3, 0.5, 0.6, 0.65, 0.7)$ and $\boldsymbol{\pi}^{\mathbf{T}} = (0.05, 0.1, 0.15, 0.2, 0.25)$ and ESS=0.9. Let $\boldsymbol{b}_{E,proposed} = (b_{E,0}, b_{E,1}, b_{E,2}, b_{E,3}, b_{E,4}, b_{E,5})$, $\boldsymbol{\sigma}_{E,proposed} = (\sigma_{E,0}, \sigma_{E,1}, \sigma_{E,2}, \sigma_{E,3}, \sigma_{E,4}, \sigma_{E,5})$, $\boldsymbol{b}_{T,proposed} = (b_{T,0}, b_{T,1}, b_{T,2}, b_{T,3})$ and $\boldsymbol{\sigma}_{T,proposed} = (\sigma_{T,0}, \sigma_{T,1}, \sigma_{T,2}, \sigma_{T,3})$ denote the prior hyperparameters for the proposed design. The elicited prior mean and chosen ESS give $\boldsymbol{b}_{E,proposed} = (0.57, 1.74, 0, 0, 0, 0)$, $\boldsymbol{\sigma}_{E,proposed} = (2.36, 2.12, 0.2, 2.5, 2.5, 0.2)$, $\boldsymbol{b}_{T,proposed} = (-4.44, 1.03, 0, 0)$ and $\boldsymbol{\sigma}_{T,proposed} = (2.62, 2.52, 2.5, 2.5)$. Additionally, we set $p_{E,3} = p_{E,4} = p_{E,5} = p_{T,2} = p_{T,3} = 0.5$. Using these hyperparameters and the utility function described in Section 3.1 of the main paper, the prior predictive probability of dose level 1 through 5 being the OBD is $(0.14, 0.17, 0.20, 0.24, 0.25)$. The prior probability of efficacy for each dose level is $(0.41, 0.51, 0.58, 0.63, 0.65)$ and the prior probability of toxicity for each dose level is $(0.15, 0.13, 0.15, 0.18, 0.21)$.

Since prior hyperparameters are calibrated assuming no subgroup effect, the prior hyperparameters for the two comparison designs given in this paper are determined in this process. Let $\boldsymbol{b}_{E,comparison} = (b_{E,0}, b_{E,1}, b_{E,2})$, $\boldsymbol{\sigma}_{E,comparison} = (\sigma_{E,0}, \sigma_{E,1}, \sigma_{E,2})$, $\boldsymbol{b}_{T,comparison} = (b_{T,0}, b_{T,1})$ and $\boldsymbol{\sigma}_{T,comparison} = (\sigma_{T,0}, \sigma_{T,1})$ denote the prior hyperparameters for the comparison designs. Using the same elicited prior means and ESS gives $\boldsymbol{b}_{E,comparison} = (0.57, 1.74, 0)$, $\boldsymbol{\sigma}_{E,comparison} = (2.36, 2.12, 0.2)$, $\boldsymbol{b}_{T,comparison} = (-4.44, 1.03)$ and $\boldsymbol{\sigma}_{T,comparison} = (2.62, 2.52)$. For the two comparison designs the prior predictive probability of dose level 1 through 5 being the OBD is $(0.10, 0.14, 0.21, 0.26, 0.28)$ for the utility function described previously. The prior probability of efficacy for each dose is $(0.38, 0.52, 0.61, 0.67, 0.70)$ and the prior probability of toxicity for each dose is $(0.09, 0.07, 0.09, 0.12, 0.15)$.

It is possible, as is the case for this set of hyperparameters and the set for the proposed design, for outcome probabilities to be non-monotone. As the absolute value of the standardized dose increases, the variance of the outcome probability's distribution will also increase. When all of the target outcome probabilities are near 0.5, the prior outcome probability is likely to be monotone because the outcome probabilities' distributions will be roughly symmetric. However, when the target outcome probabilities are near 0 or 1, non-monotonicity is likely because the outcome probabilities' distributions are skewed and, therefore, the mean is affected by the increased variance for standardized doses farther from 0.

2.1.2 | Simulation Scenarios

In the first 7 scenarios, efficacy and toxicity are independent. Figure 2 shows the true dose-efficacy curve, dose-toxicity curve, and subgroup-specific OBD for each of the simulation scenarios. The dose-efficacy and dose-toxicity models are of the same

form as that described in Section 2.1 of the main paper for scenarios 1 through 6, but differ for scenario 7. For scenario 7, efficacy and toxicity probabilities are selected for each dose and subgroup without following a specific model form.

In scenarios 1, 5 and 6, the dose-efficacy and dose-toxicity curves are the same for the two subgroups causing the OBD to also be the same. The OBD for scenario 1 is the middle dose level and is the lowest and highest dose levels for scenarios 5 and 6, respectively. The dose-efficacy curve differs for the two subgroups in scenario 2 making the OBD for the small tumor subgroup two levels higher than the OBD for the large tumor subgroup. The dose-toxicity curve differs for the two subgroups in scenario 3. Since the dose-toxicity curve for the large tumor subgroup is so steep, there is no dose that satisfies both of the toxicity and efficacy thresholds for that subgroup and, therefore, there is no OBD for the large tumor subgroup. In scenario 4, the true dose-efficacy and dose-toxicity curve vary by subgroup, but they are nearly parallel making the OBD for the two subgroups the same. In contrast, the difference by subgroup in the dose-efficacy and dose-toxicity curves in scenario 7 causes the OBD for the large tumor subgroup to be the lowest dose while the OBD for the small tumor subgroup is the highest dose.

In scenario 8, the two outcomes are positively correlated. This scenario uses the same marginal dose-efficacy and dose-toxicity curves as scenario 4, but toxicity and efficacy are positively correlated, leading to a higher chance of efficacy given toxicity occurred, as seen in Figure 2. The true OBD for both subgroups is 0.6.

2.2 | Additional Results for Primary Simulation

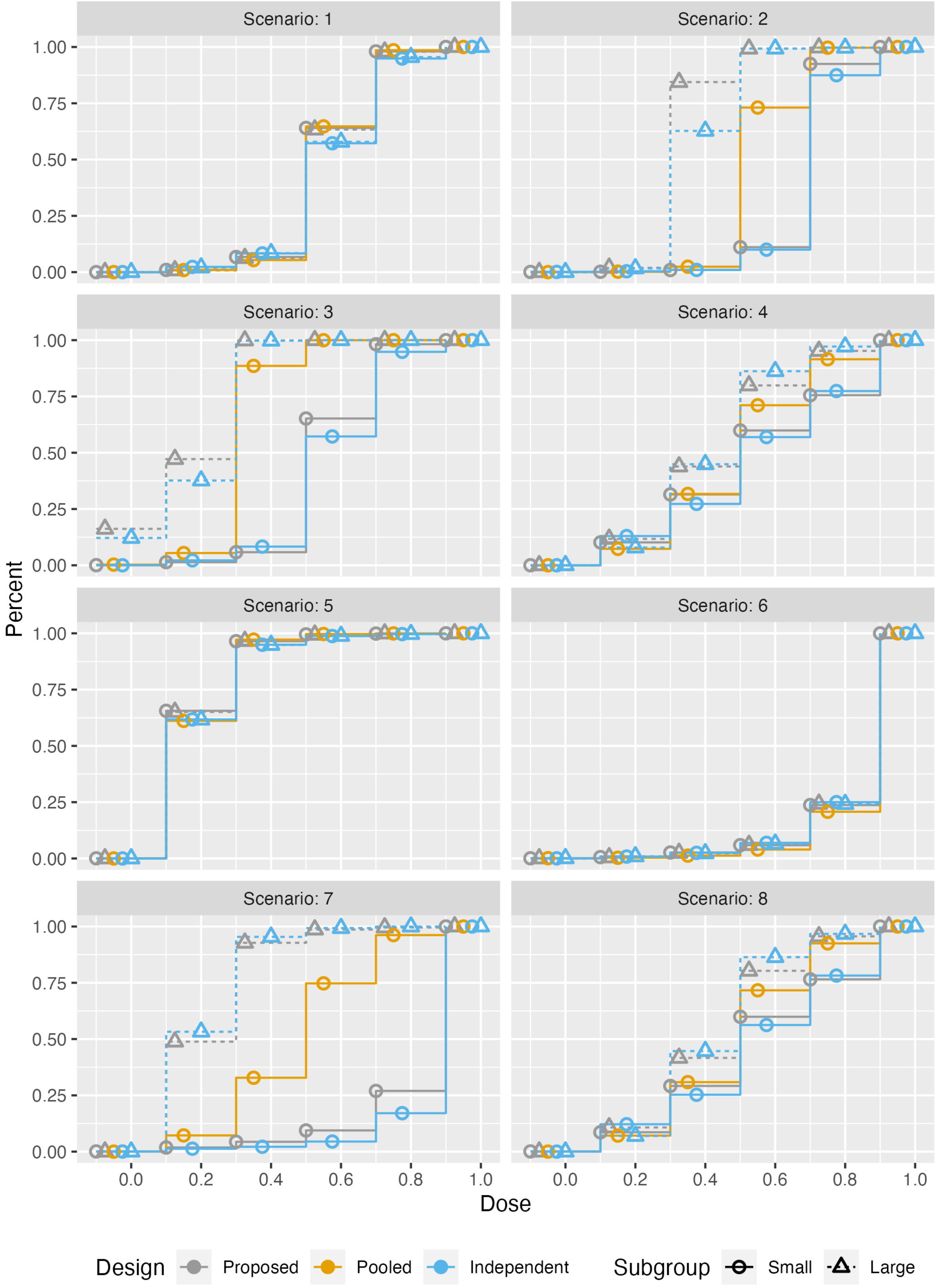

Supplemental Figure 1 Empirical cumulative distribution functions for selecting each dose as the OBD for each approach and subgroup, by scenario. Each line corresponds to a different subgroup-design combination. Since the two subgroups will always give the same result for the pooled approach, a single line is used to present those results. The symbols, circles and triangles, are used to indicate the value of the cumulative distribution function at the corresponding dose.

		Average Number Participants			Average Proportion Toxicities		
			Subgroup			Subgroup	
Scenario	Method	Overall	Small	Large	Overall	Small	Large
1	Proposed	60.00	30.00	30.00	0.19	0.19	0.19
	Pooled	60.00	30.00	30.00	0.18	0.18	0.18
	Independent	60.00	30.00	30.00	0.19	0.19	0.19
2	Proposed	59.98	29.98	30.00	0.17	0.27	0.06
	Pooled	60.00	30.00	30.00	0.15	0.15	0.15
	Independent	59.99	29.99	30.00	0.18	0.26	0.09
3	Proposed	57.76	29.98	27.77	0.26	0.18	0.35
	Pooled	59.88	29.85	29.94	0.29	0.05	0.54
	Independent	58.62	30.00	28.62	0.26	0.19	0.34
4	Proposed	59.99	29.99	30.00	0.22	0.18	0.27
	Pooled	60.00	30.00	30.00	0.23	0.15	0.30
	Independent	60.00	30.00	30.00	0.21	0.17	0.25
5	Proposed	59.99	29.99	30.00	0.19	0.19	0.19
	Pooled	60.00	30.00	30.00	0.19	0.19	0.19
	Independent	60.00	30.00	30.00	0.21	0.21	0.21
6	Proposed	59.98	29.98	29.99	0.21	0.21	0.22
	Pooled	59.95	29.98	29.98	0.22	0.22	0.22
	Independent	59.99	29.99	30.00	0.19	0.19	0.19
7	Proposed	59.98	29.98	30.00	0.19	0.12	0.25
	Pooled	60.00	30.00	30.00	0.27	0.08	0.46
	Independent	60.00	30.00	30.00	0.17	0.11	0.23
8	Proposed	59.98	29.98	30.00	0.22	0.18	0.27
	Pooled	60.00	30.00	30.00	0.23	0.15	0.30
	Independent	60.00	30.00	30.00	0.21	0.17	0.25

Supplemental Table 4 Average number of participants treated per trial and proportion of DLTs per trial.

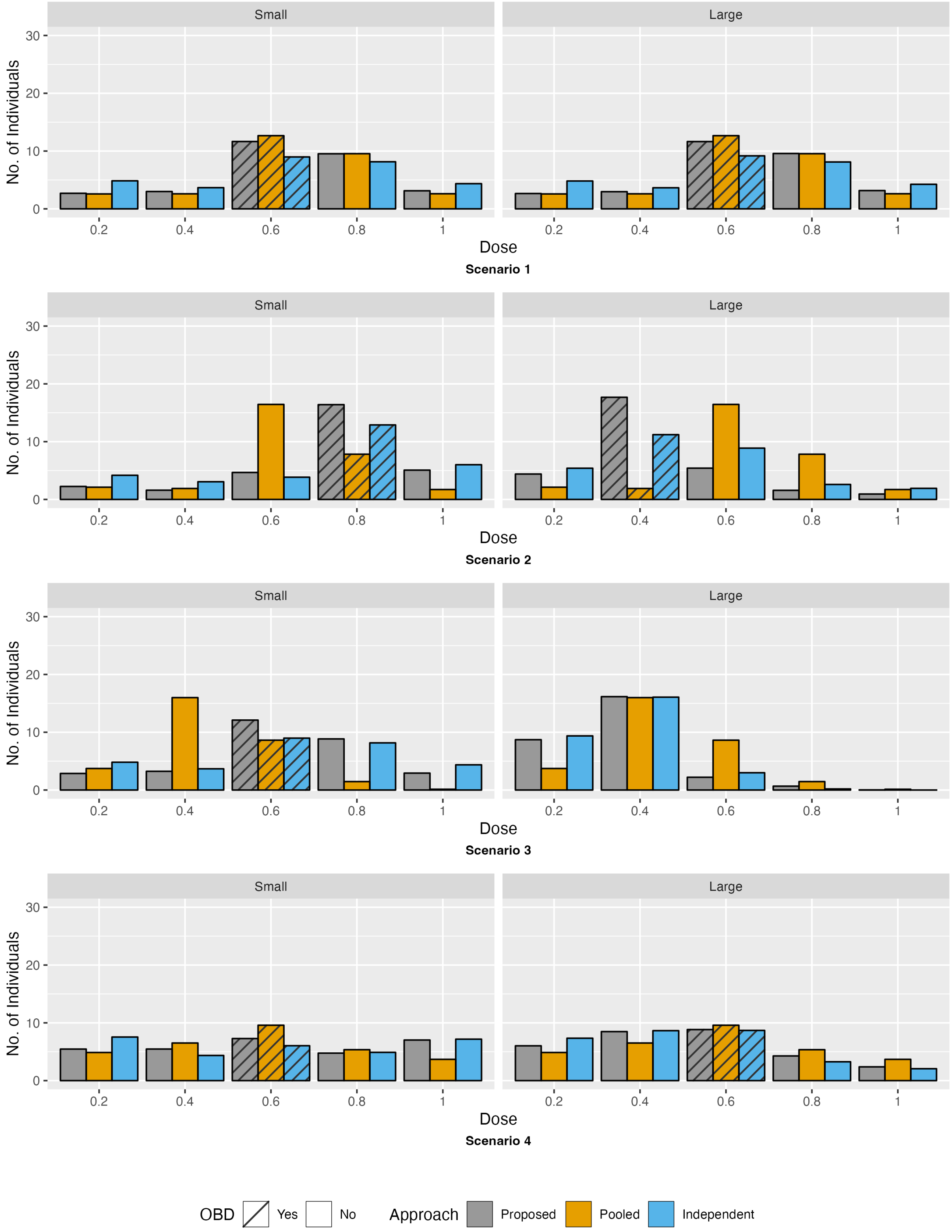

Supplemental Figure 2 Average number of individuals treated at each dose for each subgroup and approach, scenarios 1-4. Each scenario is presented in a different row. The gray bars show results from the proposed approach, the orange bars show results from the pooled approach and the blue bars show results from the independent approach. The small tumor subgroup results are given on the left and the large tumor subgroup results are given on the right.

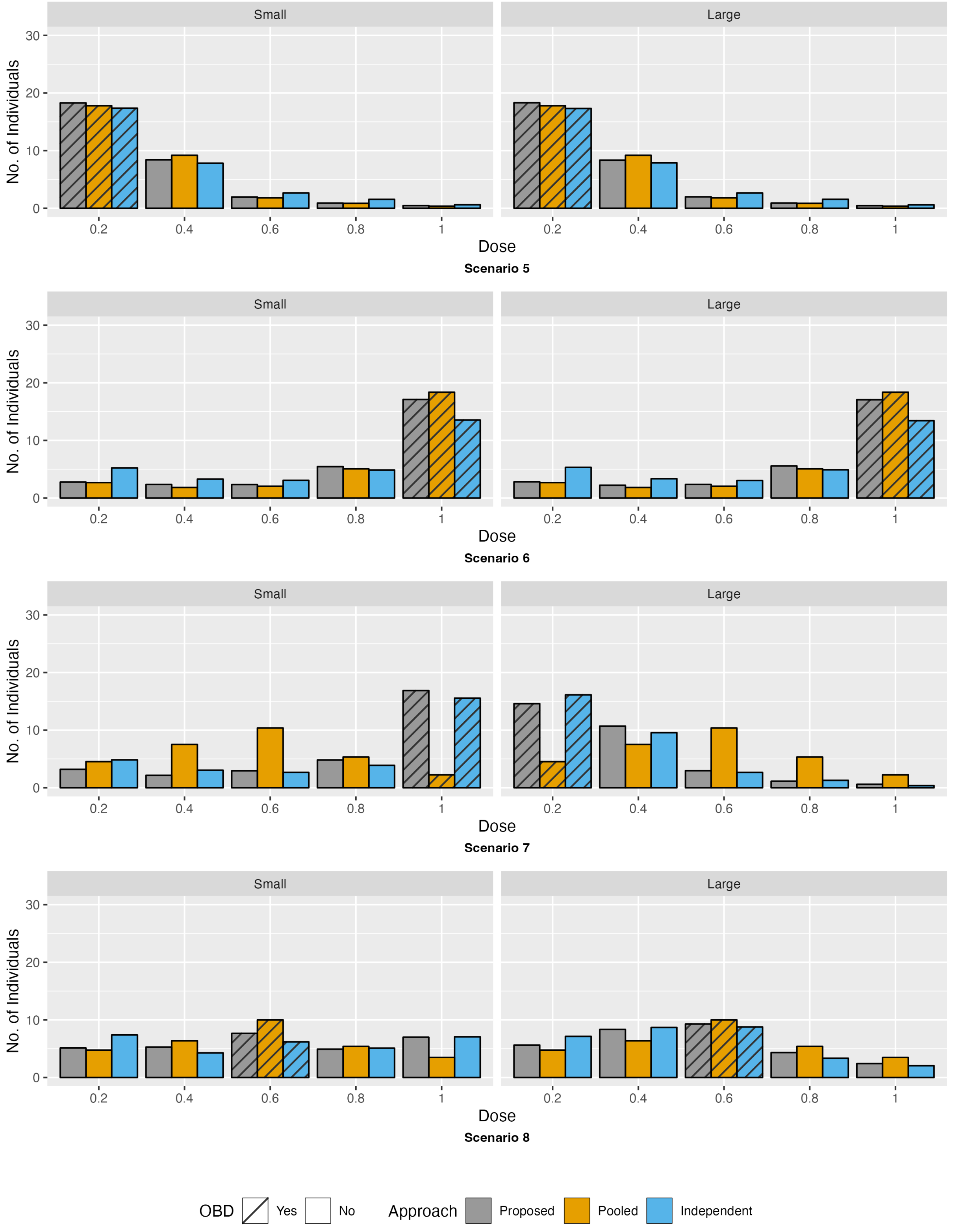

Supplemental Figure 3 Average number of individuals treated at each dose for each subgroup and approach, scenarios 5-8. Each scenario is presented in a different row. The gray bars show results from the proposed approach, the orange bars show results from the pooled approach and the blue bars show results from the independent approach. The small tumor subgroup results are given on the left and the large tumor subgroup results are given on the right.

2.3 | Sensitivity Analysis for Subgroup Combinations

In this section, we present the results of a sensitivity analysis for unrestricted subgroup combinations in each cohort. The proposed design suggests using one individual from each subgroup in each cohort enrolled; however, this is not always feasible and we would like to use participants as efficiently as possible. Thus, we conduct a sensitivity analysis for scenarios 1 through 8 which allows cohorts to be comprised of any combination of subgroups.

These unrestricted cohorts are still made up of two individuals and we require that the trial plans to enroll an equal number of individuals from each of the two subgroups. As such, enrollment order for each trial was determined by randomly assigning an entrance order to the 60 individuals (30 per subgroup). The first cohort was comprised of the individuals with entrance order 1 and 2, the second cohort was comprised of the individuals with entrance order 3 and 4, and so on until enrollment was closed for one or both subgroups. If enrollment was closed for both subgroups, the trial ended early. If enrollment was closed for one subgroup, the next cohort must have been comprised of two individuals from the other subgroup and cohorts would have this same composition until enrollment for the first subgroup was reopened or 30 individuals from the second subgroup had been enrolled. It is possible that outcomes were only observed in one subgroup during the rule-based run-in and that the transition to the model-based dose assignment depended solely on that subgroup's data.

	Recommended dose for Small Tumor Group						Recommended dose for Large Tumor Group					
Scenario	0	0.2	0.4	0.6	0.8	1	0	0.2	0.4	0.6	0.8	1
1	0.00	0.01	0.05	0.58	0.34	0.02	0.00	0.01	0.05	0.58	0.34	0.02
2	0.00	0.00	0.01	0.09	0.82	0.08	0.00	0.02	0.82	0.15	0.00	0.00
3	0.00	0.01	0.04	0.60	0.33	0.02	0.16	0.30	0.54	0.00	0.00	0.00
4	0.00	0.09	0.20	0.31	0.18	0.22	0.00	0.11	0.32	0.37	0.16	0.04
5	0.00	0.65	0.31	0.03	0.00	0.00	0.00	0.67	0.31	0.03	0.00	0.00
6	0.00	0.01	0.01	0.03	0.17	0.78	0.00	0.00	0.01	0.03	0.18	0.77
7	0.00	0.02	0.02	0.05	0.17	0.74	0.00	0.49	0.44	0.06	0.01	0.00
8	0.00	0.07	0.20	0.31	0.18	0.24	0.00	0.10	0.31	0.37	0.17	0.05

Supplemental Table 5 Probability of recommending each dose as the OBD when using the proposed approach but allowing any combination of subgroups in enrollment cohorts.

The results presented in Supplemental Table 5 are only slightly different from those presented for elicitation 1 in Supplemental Table 3 which indicates that enrolling one individual per subgroup in each cohort is not crucial to the performance of the proposed design with respect to correctly selecting the OBD. Additionally, Supplemental Table 6, when compared to Supplemental Table 4, shows that the average proportion of DLTs may be slightly elevated for some scenarios but mostly remains the same when taking the unrestricted cohort composition approach. Additionally, the average number of participants treated per trial for each subgroup differed minimally and the per subgroup and overall results were similar to those presented in Supplemental Table 4 (results not shown).

	Average Proportion Toxicities		
		Subgroup	
Scenario	Overall	Small	Large
1	0.18	0.18	0.19
2	0.17	0.27	0.06
3	0.26	0.18	0.35
4	0.22	0.18	0.27
5	0.19	0.19	0.19
6	0.21	0.21	0.21
7	0.19	0.12	0.25
8	0.23	0.18	0.27

Supplemental Table 6 Average proportion of DLTs per trial, when using the proposed approach but allowing any combination of subgroups in enrollment cohorts.

2.4 | Sensitivity Analysis for Rule-based Run-in

In this section, we present the results of a sensitivity analysis for a fully model-based dose-assignment approach (not using the rule-based run-in). The proposed design suggests using a rule-based run-in to ensure the first dose assignment is always the lowest dose of the trial; restrict the speed of dose-escalation to prevent overly toxic doses from being assigned when there is insufficient data; keep dose-escalation from differing in the two subgroups when there is insufficient information to determine if the OBD differs for the subgroups; and escalate the initial dose during a period where the dose-assignment model would be unstable and give poor estimates. However, concerns may arise regarding the loss of efficiency when using a rule-based run-in.

	Recommended dose for Small Tumor Group						Recommended dose for Large Tumor Group					
Scenario	0	0.2	0.4	0.6	0.8	1	0	0.2	0.4	0.6	0.8	1
1	0.00	0.01	0.05	0.57	0.35	0.02	0.00	0.01	0.05	0.57	0.36	0.02
2	0.00	0.00	0.00	0.08	0.84	0.08	0.00	0.02	0.82	0.16	0.00	0.00
3	0.00	0.01	0.04	0.59	0.34	0.02	0.17	0.30	0.53	0.00	0.00	0.00
4	0.00	0.08	0.20	0.29	0.19	0.29	0.00	0.10	0.32	0.39	0.19	0.05
5	0.00	0.68	0.37	0.03	0.00	0.00	0.00	0.69	0.36	0.04	0.00	0.00
6	0.00	0.00	0.01	0.03	0.19	0.76	0.00	0.00	0.01	0.03	0.19	0.76
7	0.00	0.02	0.02	0.05	0.15	0.76	0.01	0.46	0.46	0.05	0.01	0.00
8	0.00	0.07	0.19	0.31	0.18	0.27	0.00	0.08	0.30	0.39	0.19	0.04

Supplemental Table 7 Probability of recommending each dose as the OBD when using the proposed approach but omitting the rule-based run-in.

The results presented in Supplemental Table 7 differ marginally from those presented for elicitation 1 in Supplemental Table 3 which indicates that use of the rule-based run-in has little effect on the ability of the design to adequately explore the range of doses. Additionally, Supplemental Table 8, when compared to Supplemental Table 4, shows that the average proportion of DLTs is elevated when the rule-based run-in is omitted and model-based dose-assignment is used for the entire trial. Notably, there is

also no major gain in efficiency as the average number of participants enrolled is approximately the same with or without the rule-based run-in.

		Average Number Participants			Average Proportion Toxicities		
			Subgroup			Subgroup	
Scenario	Method	Overall	Small	Large	Overall	Small	Large
1	Proposed	59.98	29.99	30.00	0.20	0.20	0.20
	Pooled	60.00	30.00	30.00	0.19	0.19	0.19
	Independent	60.00	30.00	30.00	0.21	0.21	0.21
2	Proposed	59.97	29.97	30.00	0.17	0.29	0.06
	Pooled	60.00	30.00	30.00	0.17	0.17	0.17
	Independent	60.00	30.00	30.00	0.19	0.28	0.10
3	Proposed	57.52	29.98	27.54	0.27	0.19	0.37
	Pooled	59.80	29.90	29.90	0.31	0.06	0.57
	Independent	58.10	30.00	28.10	0.29	0.21	0.38
4	Proposed	59.97	29.99	29.98	0.24	0.19	0.28
	Pooled	59.99	29.99	29.99	0.25	0.17	0.33
	Independent	59.99	30.00	29.99	0.23	0.20	0.27
5	Proposed	59.86	29.93	29.94	0.20	0.20	0.20
	Pooled	59.91	29.95	29.95	0.20	0.20	0.20
	Independent	59.95	29.97	29.98	0.22	0.23	0.22
6	Proposed	59.98	29.99	29.99	0.23	0.23	0.23
	Pooled	59.99	30.00	30.00	0.23	0.23	0.23
	Independent	59.99	30.00	30.00	0.21	0.21	0.21
7	Proposed	59.75	29.99	29.75	0.19	0.13	0.27
	Pooled	59.99	30.00	30.00	0.30	0.09	0.50
	Independent	59.98	30.00	29.98	0.19	0.12	0.25
8	Proposed	59.94	29.97	29.96	0.24	0.19	0.29
	Pooled	59.99	30.00	30.00	0.24	0.16	0.33
	Independent	59.99	30.00	29.99	0.23	0.20	0.27

Supplemental Table 8 Average number of participants treated per trial and proportion of DLTs per trial.

References

1. Thall PF, Herrick RC, Nguyen HQ, Venier JJ, Norris JC. Effective sample size for computing prior hyperparameters in Bayesian phase I–II dose-finding. *Clinical Trials* 2014; 11(6): 657–666. doi: 10.1177/1740774514547397

2. Gelman A. Prior Choice Recommendations. 2021.

□
